# Maternal Diet, Lifestyle Factors, and Gestational Weight Gain: A Single-Center Case–Control Study in Hungary

**DOI:** 10.3390/nu18091403

**Published:** 2026-04-29

**Authors:** Edit Paulik, Anita Sisák, Anna Szolnoki, Evelin Olteán-Polanek, Márió Gajdács, Regina Molnár, Andrea Szabó, Gábor Németh, Hajnalka Orvos

**Affiliations:** 1 Department of Public Health, Albert Szent-Györgyi Medical School, University of Szeged, 6720 Szeged, Hungary; gajdacs.mario@med.u-szeged.hu (M.G.); molnar.regina@med.u-szeged.hu (R.M.); szabo.andrea@med.u-szeged.hu (A.S.); 2 Doctoral School of Experimental and Preventive Medicine, University of Szeged, 6720 Szeged, Hungary; 3 Department of Obstetrics and Gynecology, Bajcsy-Zsilinszky Hospital, 1106 Budapest, Hungary; szolnoki.anna97@gmail.com; 4 Ministry of Foreign Affairs and Trade, 1027 Budapest, Hungary; evelin.polanek@gmail.com; 5 Department of Obstetrics and Gynecology, Albert Szent-Györgyi Medical School, University of Szeged, 6725 Szeged, Hungary; nemeth.gabor@med.u-szeged.hu (G.N.); kokutine.orvos.hajnalka@med.u-szeged.hu (H.O.)

**Keywords:** case–control study, gestational weight gain, maternal diet, preterm birth

## Abstract

Background/Objectives: Preterm birth (PTB) is a major public health concern worldwide, which may lead to detrimental maternal and neonatal outcomes. Maternal nutritional status, gestational weight gain (GWG), and lifestyle factors are potentially modifiable determinants of adverse pregnancy outcomes. This study examined the association between PTB and maternal GWG and assessed whether maternal dietary habits and lifestyle factors were related to GWG in women delivering preterm versus at term. Methods: A retrospective case–control study was conducted at a tertiary center in Hungary (MANOR Study, 2019). The case group included *n* = 100 women with PTB, while *n* = 200 matched term deliveries served as controls (1:2 ratio). Data were collected using a self-administered questionnaire and medical records. Pre-pregnancy body mass index (BMI) was categorized using standard definitions, while GWG was classified as inadequate, recommended, or excessive according to the US 2009 Institute of Medicine guidelines. A 7-item dietary index score was calculated based on gestational dietary habits. Results: Pre-pregnancy BMI distribution did not considerably differ between groups (*p* > 0.05); over one-third of women in both groups were overweight or had obesity (38.7% vs. 36.7%). Previous PTB (*p* < 0.001) and gestational hypertension (GHT) (*p* = 0.003) were more common among current PTB cases, while smoking, alcohol consumption, and gestational diabetes mellitus (GDM) showed negligible differences (*p* > 0.05)—28.0% of cases, and 34.5% of controls were classified as having healthy dietary habits, based on the dietary index score calculated. Inadequate GWG was more prevalent among PTB cases (49.0% vs. 26.8%), whereas excessive GWG was less frequent among cases (21.9% vs. 38.4%). Being within the recommended GWG range and the manifestation of gestational hypertension were associated with lower (aOR: 0.39; 95% CI: 0.18–0.87; *p* = 0.020) and higher (aOR: 3.43; 95% CI: 1.44–8.19; *p* = 0.005) odds of PTB, respectively. Conclusions: Inadequate GWG was more common in PTB, while excessive GWG was more frequent in term pregnancies. Fast-food consumption was associated with excessive GWG among term births. Optimizing GWG and improving maternal diet quality should be included as key, cross-cutting interventions targeting the improvement of antenatal care.

## 1. Introduction

Preterm birth (PTB) is characterized by a delivery occurring between the 24th and 37th completed weeks of gestation [1]. According to the World Health Organization (WHO), PTBs affect 4–16% of all live births, and complications related to prematurity are one of the leading causes of death in children under 5 years of age, associated with ~900,000 deaths in 2019 alone [1]; according to the U.S. Centers for Disease Control and Prevention (CDC) data, around 14% of infant deaths are linked to PTB or low birth weight (LBW) [2,3]. Furthermore, 2021 estimations from the Global Burden of Disease (GBD) study highlighted that—while high Socio-Demographic Index (SDI) regions managed to reduce their annual incidence of PTB by 9.6% (−11.45% to −7.79%)—its incidence has been increased by 43.1% (+40.17 to +46.09%) in low SDI regions, in the last 30 years, constituting a sizable burden of disability-adjusted life-years (DALYs) globally [4]. In Hungary, the rate of PTBs has remained constant between 8 and 9% among live births since the 1990s [5], even in 2023, the rate was 8.2% and 8.4% nationwide, and in the Southern region of Hungary, respectively [6].

Advances in medical science have contributed to a reduction in infant mortality; however, PTBs may have a detrimental impact on population morbidity without leading to an infant’s death directly: among preterm infants who survive, the emergence of a wide range of long-term adverse outcomes has been documented, including cerebral palsy, delayed cognitive development, sensory and motor impairments [2], developmental disorders of the respiratory system, cardiovascular anomalies, obesity [7] and diabetes mellitus [8]. As the potential sequelae associated with PTBs may impact an individual’s entire life course, sustained, cross-cutting preventive efforts are necessary [9]. Depending on the length and severity of PTB-related consequences, impacted families may carry considerable direct and indirect economic and psychosocial burden [2].

Numerous maternal factors have been identified as potential risk factors for PTB, including maternal age (under 18 or over 35 years) [10,11], chronic diseases (e.g., hypertension, pre-gestational diabetes) [12], uterine anomalies [13], the lack of prenatal care [14], multiple pregnancies [15] and relevant lifestyle factors, such as smoking [16,17], substance use [18], alcohol consumption [19], chronic psychosocial stress [20], lack of leisure-time physical activity [21], nutritional deficiencies [22,23,24], dietary habits, and considerable weight changes during pregnancy [25]. Nonetheless, in the majority of cases, the principal cause of PTB has remained unidentified, or may involve multiple causal contributing factors [26].

Inadequate gestational weight gain (GWG) and poor nutritional habits may be key contributors to the increased risk of premature delivery [27]. In the context of gestational weight status, lower pre-pregnancy maternal body mass index (BMI) has also been associated with PTB [26]. Women with a BMI < 20 kg/m^2^ have had nearly a four-fold increase in the risk of PTB compared to women with higher BMI [25].

A meta-analysis published by Liu et al. reports that pre-pregnancy BMI outside the recommended range (i.e., under- or overweight/obesity) is associated with adverse neonatal outcomes, including PTB, small-for-gestational age (SGA), large-for-gestational age (LGA), macrosomia, and neonatal asphyxia, indicating that both the pre-pregnancy BMI and GWG play key roles in perinatal outcomes [28].

According to the guidelines published by the Institute of Medicine (IOM) (Washington, DC, USA, 2009) [29], the recommended GWG for singleton pregnancies are determined by women’s pre-pregnancy BMI categories, i.e., women within the normal pre-pregnancy BMI (18.5–24.9 kg/m^2^) range are advised to gain between 11.5 and 16.0 kg during pregnancy in case of a full-term delivery (40 weeks of gestation). In comparison, for women being underweight (BMI < 18.5 kg/m^2^) in the pre-pregnancy period, the recommended weight gain is higher (between 12.5 and 18.0 kg), while for overweight (BMI 25.0–29.9 kg/m^2^) and women with obesity (BMI ≥ 30.0 kg/m^2^), the recommended optimal weight gains are lower, between 7.0 and 11.5 kg and 5.0–9.0 kg, respectively. GWG below, within, or above these ranges is classified as “inadequate,” “recommended,” or “excessive” GWG [29,30]. A systematic review and meta-analysis, corresponding to a cohort from 10 countries, by Goldstein et al. [31] state that 23% of women gained less and 47% gained more weight than the US 2009 IOM recommendations described above. GWG below guidelines is associated with higher risks of PTB and SGA infants, whereas GWG above guidelines is associated with higher risks of cesarean section, LGA infants, and slightly lower risk of PTB [28,31]. Data from countries at all income levels further underscore that GWG below guideline recommendations is linked to increased risks of PTB, SGA, and LBW. However, findings about excessive GWG and the likelihood of PTB are inconsistent across published studies [31,32,33].

Nutritional factors have also been linked to several maternal, fetal, and infant health outcomes. A balanced diet is essential during pregnancy, as studies have shown that it may decrease the risk of gestational diabetes mellitus (GDM), gestational hypertension (GHT), LBW (<2500 g), and PTB [34,35]. In addition, an unhealthy maternal diet may have considerable long-term influences on the health and well-being of the child, contributing to poor cognitive development, elevating the risk of obesity, allergies, asthma, diabetes, and hypertension later in life [36,37,38]. In addition to the above, interventional studies suggest that lifestyle interventions during pregnancy may limit excessive GWG and reduce the risk of GDM, GHT, LGA, and, in some analyses, PTB, although the evidence related to this outcome is not robust [39,40].

During pregnancy, the overall dietary patterns appear to have a greater impact on health outcomes than the presence of individual nutrients. Systematic reviews and meta-analyses corresponding to both developing and developed regions have found that healthy dietary behaviors (including high consumption of vegetables, fruits, whole grains, legumes, dairy products, and fish) and limited consumption of the components of the “Western diet” (i.e., energy-dense, highly processed foods) and sugar-sweetened soft drinks reduce the risk of GDM, GHT, and adverse pregnancy outcomes [34,35,41]. The underlying mechanism—suggested by the literature—is that diets rich in ultra-processed, energy-dense foods, fats, and sugars are characterized by high energy yet low nutritional quality, linked to excessive GWG and inadequate infant birth weight [42].

Several studies have highlighted that pre-pregnancy BMI, GWG, and maternal lifestyle are all important determinants of infant health, and they may act as modifiable risk factors in the prevention of PTB and perinatal complications [28,31]. However, a crucial knowledge gap has remained: the strength of the associations has been highly inconsistent across the existing literature, likely due to variations in regional demographics, population-level health status, and clinical practices. As current evidence has lacked granularity or local context, it results in a bottleneck that impedes the effective planning and implementation of primary preventive interventions in specific local contexts during the gestational period. Thus, the objective of our present study was to assess this gap: specifically, we aimed to assess the relationship between PTB and gestational weight change, while simultaneously investigating whether prior obstetric events, chronic maternal diseases, and certain lifestyle factors were associated with maternal weight trajectories during pregnancy among women who delivered preterm versus those who delivered at term. The aim of this study was to contribute to the evidence base necessary for more precise, localized prenatal care protocols.

The following working hypotheses directed our analyses: (i) women characterized by GWG below or above guideline-recommended ranges are more likely to experience PTB, (ii) women characterized by GHT, GDM and prior PTB are more likely to experience PTB, (iii) women who consumed tobacco and alcohol during gestation are more likely to experience PTB, (iv) women reporting unhealthy dietary patterns and no physical activity during gestation are more likely to report GWG below or above guideline-recommended ranges, (v) women who consumed tobacco and alcohol during gestation are more likely to report GWG below or above guideline-recommended ranges.

## 2. Materials and Methods

### 2.1. Study Design

A retrospective, questionnaire-based, matched case–control observational study was conducted at the Department of Obstetrics and Gynecology, Albert Szent-Györgyi Clinical Center (CC), and the Department of Public Health, Albert Szent-Györgyi Faculty of Medicine, University of Szeged, where women who gave birth at the CC were considered as the pool of potential study participants. The CC is a tertiary-care teaching hospital, providing access to primary and specialized health facilities at both the county (Csongrád-Csanád) and regional (Southern Great Plain of Hungary) levels. The current study was carried out as a part of a larger investigation entitled “Quantifying MAternal Non-Obstetrical Risk Factors for Preterm Birth—Retrospective and Prospective Study” (the MANOR Study). The retrospective study was conducted between March and December 2019.

### 2.2. Study Participants, Sample Size, Inclusion and Exclusion Criteria

**Figure 1:**
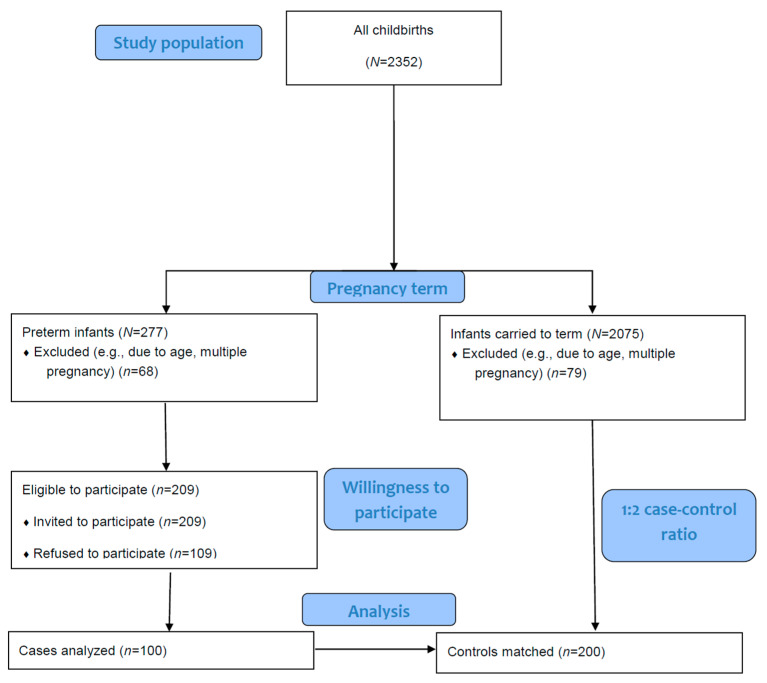
Flowchart of the sample selection process.

Participants of the study included the following two populations: cases (women with PTB), i.e., women who were: (i) willing to participate in the study, and had provided informed consent, (ii) delivered before the 37th week of gestation, (iii) were at least 18 years of age, and (iv) were singleton pregnancies. Women with PTB who refused participation in the study or had any barring characteristics (e.g., twin pregnancies) were excluded. During the study period, *N* = 2352 newborns were born, out of which *N* = 277 (11.78%) were preterm infants. Considering the inclusion and exclusion criteria, *N* = 209 women-infant dyads were eligible to participate, out of which *n* = 100 were ultimately included in our analysis (Figure 1). With a 1:2 case–control ratio in mind, in post hoc power analyses, this sample size provided 80% power to detect that changes in a magnitude of ~1.5 in a quantitative variable, assuming a 20% exposure prevalence among controls, and a two-sided α = 0.05.

The second population—i.e., the controls—were women who were: (i) willing to participate in the study, and had provided informed consent, (ii) delivered after the 37th completed week of gestation (term births), (iii) were at least 18 years of age, (iv) had singleton pregnancies. The study employed a frequency matching approach with a 1:2 case-to-control ratio (i.e., resulting in *n* = 200 controls out of the *N* = 2075 eligible women, before applying the exclusion criteria); matching was carried out based on maternal age (±2 years) and parity, without adjusting for additional confounding factors. Exclusion criteria were similarly applied to cases as well.

### 2.3. Data Collection, Outcome Measures

Data collection was based on three types of data sources: (i) a self-administered, structured, paper-based questionnaire, (ii) medical documentation, and (iii) results of maternal serum and umbilical cord-blood vitamin D laboratory analyses. Cases and controls completed the questionnaires during their postpartum hospital stay. If the participant needed to take part in a medical exam/consultation, then questionnaire-based data collection was suspended, which was resumed after the patient completed the required assessment.

The questionnaire included items related to: (i) sociodemographic characteristics, (ii) lifestyle-related factors, (iii) contraception, planning of pregnancy, number of previous pregnancies, previous pregnancy complications (e.g., miscarriage) and PTB, and (iv) general health status. Sociodemographic data collected in the present analysis included maternal age, level of educational attainment (primary and secondary school were classified as lower vs. college and university as higher), type of residence (county town, town, and village), and relationship status (unmarried and widowed were classified as single vs. married and living with a partner as in a relationship).

Assessment of lifestyle habits included self-reported pre-pregnancy and pre-delivery body weight, known chronic conditions during pregnancy (e.g., hypertension, GDM), behavioral risk/protective factors, such as physical activity (yes vs. no), smoking (yes vs. no), alcohol consumption (yes vs. no), following a special diet (e.g., low-sodium, GDM diet), and a 7-item questionnaire on dietary habits (i.e., consumption frequency of vegetables, fruits, fish, soft drinks, salty snacks, fast food, sweets). Based on the responses of the above food frequency item, a 0–12-point dietary index score was developed, as described by Polanek et al. (see Table 1), where higher scores indicate healthier dietary behaviors, including regular vegetable, fruit, and fish consumption, occasional or less frequent fast food, salty and sweet snack, and sugary soft drink consumption, respectively. The rationale for using this brief screening tool was: (i) the similarity of our study population characteristics (i.e., postpartum women in the same geographical region), and (ii) due to the study design, setting, and the data collection time, a more detailed dietary assessment (e.g., food frequency questionnaires with portion sizes) was impractical. Participants attaining ≥ 10 points in the diet field were considered as “healthy diet”, while scores ˂ 10 points were designated as “unhealthy diet”, respectively [43].

BMIs were calculated from self-reported pre-pregnancy weight and height, and women were categorized as underweight, normal weight, overweight, or obese, according to established BMI categories (Table 2).

GWG was analyzed according to categories described for recommended weight gain ranges defined by the IOM [29], stratified by pre-pregnancy BMI categories (see Table 2).

Additionally, the expected GWG at any gestational age was estimated using the following formula, described by Palumbo et al. [30]: expected GWG = recommended first-trimester gain + (gestational age in weeks − 13) × recommended weekly gain in the second and third trimesters [30]. Women whose GWG fell below the recommended range were classified as “inadequate”, those who were within the recommended range were categorized as “within the recommended weight gain range”, and those who exceeded the breakpoint were classified in the “excessive” category.

### 2.4. Statistical Analysis

Data collected during the analysis were entered into spreadsheets (Microsoft Excel; Microsoft Corp., Redmond, WA, USA) for data management, which were later transferred to Statistical Package for Social Sciences v.28.0 (SPSS; IBM Corp., Endicott, NY, USA) for analysis. Descriptive statistics were used to present the characteristics of the sample (frequencies (*n*) and percentages (%) for categorical variables, while means and 95% confidence intervals (95%CI) for continuous variables). Normality testing of continuous variables (e.g., dietary index) was carried out using Q-Q (quantile-quantile) diagrams and Kolmogorov–Smirnov tests. The following univariate analyses were performed: (a) to detect differences between proportions, the χ^2^-test and Fisher’s exact tests were used; (b) comparisons between groups in regard to continuous data were done using Welch *t*-tests, and Welch-ANOVA with Tukey’s post hoc tests. Furthermore, multivariable logistic regression (LR) analyses were also implemented. In Model 1, PTB experience (yes/no) was the main outcome measure, while in Model 2, excessive GWG (yes/no) was the main outcome measure; inclusion of the predictor variables was based on the specifics of the study design, potential clinical relevance, and parsimony. *p* values below 0.05 (*p* < 0.05) were considered statistically significant. During LR, the coefficients (β), standard error (SE), adjusted odds ratios (aORs), and 95% confidence intervals (95% CI) were reported.

### 2.5. Ethical Considerations

The study was conducted in accordance with the Declaration of Helsinki (1975, latest revision: 2024) [44] and national and institutional ethical standards. Ethical approval for the study protocol was obtained from the Human Institutional and Regional Biomedical Research Ethics Committee at the University of Szeged, Hungary (reference number: 256/2018-SZTE [4419]; approval date: 10 December 2018).

### 2.6. Informed Consent Statement

Before taking part in this study, participants were briefed about the nature and objectives of the study, privacy, anonymity, and confidentiality of their data; cases and controls who agreed to take part provided written informed consent before data collection. All participants were made aware that their participation in the research was voluntary, and they may withdraw from the study at any time without any consequences to their medical care. No incentives (e.g., monetary, gift, or other remuneration) were provided to participate in the study.

### 2.7. Reporting Guidelines

To ensure transparency, clarity, and consistency in the reporting of the results of our study, the present manuscript adheres to the Strengthening the Reporting of Observational Studies in Epidemiology (STROBE) guidelines for observational studies [45]; the STROBE checklist has been provided as Supplementary Material S1.

## 3. Results

### 3.1. Demographic Characteristics, Anamnestic Data, and Behavioral Factors of the Study Populations

Overall, one hundred (*n* = 100) women with PTB (i.e., cases) were included in our study, together with two hundred (*n* = 200) matched control subjects, who carried to term. The overall characteristics of the two groups are summarized in Table 3. Consistent with our sampling design and matching, the age distribution of the case and control groups showed no notable differences (*p* = 0.965); the mean age of cases and controls was 32.33 years and 32.25 years, respectively. 36.0% (*n* = 108) of women overall were ≥35 years of age. No relevant differences were observed between the two groups, by relationship status or residence, although a tendency towards higher educational attainment was observed in the control group (62.0% vs. cases, 48.0%; *p* = 0.055) (Table 3).

The distribution of pre-pregnancy BMI was also similar between the case and control groups (overweight or obese: 38.7% vs. 36.7%; *p* = 0.882). On the other hand, cases had a more frequent history of PTB (31.7% vs. controls: 9.0%; *p* < 0.001). No significant differences were observed for the frequency of smoking (*p* = 0.122), alcohol consumption (*p* = 0.110), or physical activity during pregnancy (*p* = 0.362) among cases vs. controls (Table 3). Furthermore, 15.2% of women had GDM, and 48.4% followed the GDM diet (with no significant differences between cases and controls; *p* = 0.286 and *p* = 0.231, respectively). Cases were more likely to be affected by GHT (18.4% vs. controls: 7.1%; *p* = 0.003), but not more likely to adhere to a low-sodium diet (*p* = 0.967).

### 3.2. Dietary Habits and Dietary Index Score of Cases and Controls

The responses of women with PTB and women with term deliveries (controls) regarding their dietary habits—which were used as the basis of the determination of the dietary index scores—are shown in Table 4. Daily vegetables (82.9% vs. cases: 69.4%; *p* = 0.025) and fruits (84.1% vs. 76.0%; *p* = 0.043) consumption were more common among controls, while no relevant differences were described for the frequency of fish consumption (weekly: 35.6% overall). Regarding more disadvantageous health habits, no notable disparities were identified in the frequency of eating fast foods, consuming salty snacks, sweets and soft drink daily (overall: 23.0%, 6.4%, 29.3% and 9.4%, respectively; *p* < 0.05 in all cases); however, 51.0%, 58.6% and 77.0% reported the rare (monthly or never) consumption of salty snacks, soft drinks and fast foods, respectively (Table 4).

Based on participants’ responses, mean dietary index scores were 8.29 [95%CI: 7.90–8.68] for women with PTB vs. 8.65 [95%CI: 8.37–8.93], where no relevant differences were shown between cases and controls (*p* = 0.107; overall: 8.53 [95%CI: 8.30–8.76]); in line with our previously determined criteria, they were classified as having a healthy (≥10 points) or unhealthy diets (˂10 points): accordingly, 28.0% (*n* = 28) of cases, and 34.5% (*n* = 69) of controls had healthy dietary habits (overall: 32.3% [*n* = 97] of participants were classified as healthy). Based on age, only a non-significant, but tendentious increase in mean dietary scores were identified among women with PTB (18–24 years: 7.18 [95%CI: 5.98–8.37] vs. 25–34 years: 8.29 [95%CI: 7.74–8.84] vs. ≥35 years: 8.67 [95%CI: 8.03–9.32]; *p* = 0.076), while a significant and consistent increase was shown among women who carried to term (18–24 years: 7.43 [95%CI: 6.50–8.35] vs. 25–34 years: 8.53 [95%CI: 6.50–8.35] vs. ≥35 years: 9.21 [95%CI: 8.74–9.67]; *p* = 0.002); during post hoc analyses, significant differences were identified among all age groups (*p* < 0.05 in all cases). No relevant associations were observed among dietary index scores and educational attainment (i.e., lower vs. higher) of pregnant women, either in the PTB group (8.21 [95%CI: 7.62–9.07] vs. 8.55 [95%CI: 7.90–9.37]; *p* = 0.397) or in the control group (8.34 [95%CI: 7.79–8.93] vs. 8.85 [95%CI: 8.21–9.09]; *p* = 0.093), respectively. Controls who followed the GDM diet during pregnancy had higher mean dietary scores (9.24 [95%CI: 8.95–9.67] vs. no GDM diet: 8.70 [95%CI: 8.16–8.79]; *p* = 0.005), while similar associations were not identified among cases (9.91 [95%CI: 7.63–10.35] vs. no GDM diet: 8.81 [95%CI: 6.98–9.45]; *p* = 0.266). Furthermore, no associations were identified among those following the low-sodium diet during pregnancy and mean dietary scores, in either the PTB (9.02 [95%CI: 7.23–10.08] vs. no low-sodium diet: 8.91 [95%CI: 7.15–9.82]; *p* = 0.816) or control (9.47 [95%CI: 8.22–10.03] vs. no low-sodium diet: 8.66 [95%CI: 7.98–9.81]; *p* = 0.133) groups. Finally, only non-significant tendentious differences in the dietary index scores were observed in either the cases and controls, on the basis of smoking (cases: 7.38 [95%CI: 6.87–9.04] vs. no smoking: 8.38 [95%CI: 7.16–9.45]; *p* = 0.151; controls: 7.43 [95%CI: 7.01–9.17] vs. no smoking: 8.69 [95%CI: 8.02–9.11]; *p* = 0.111), consumption of alcohol (cases: 7.60 [95%CI: 7.03–8.94] vs. no alcohol: 8.31 [95%CI: 7.23–9.34]; *p* = 0.220; controls: 8.52 [95%CI: 7.99–9.56] vs. no alcohol: 8.67 [95%CI: 7.64–9.39]; *p* = 0.355), or taking part in physical activity (cases: 8.59 [95%CI: 7.43–9.83] vs. no physical activity: 7.96 [95%CI: 7.11–9.38]; *p* = 0.067; controls: 8.81 [95%CI: 8.12–9.56] vs. no physical activity: 8.47 [95%CI: 7.85–9.32]; *p* = 0.116) during pregnancy.

### 3.3. Association Between Pregnancy Weight Gain, Pregnancy Term, and Other Obstetric Outcomes and Events

Out of the pregnant women participating in our study, less than one-third (27.9%; *n* = 84) were characterized by the recommended GWG range, as described by IOM criteria previously. When comparing cases and controls (*p* = 0.015), women who had inadequate weight gain were more common among PTBs (39.6% vs. term pregnancies: 25.8%), while adequate GWG was comparatively less common in PTBs (18.8% vs. term pregnancies: 32.3%); on the other hand, rates of excessive GWG were similar in both groups (41.7% vs. 41.9%).

No associations between GWG categorization and mean dietary scores were identified among women with PTB (inadequate: 8.48 [95%CI: 7.778–9.19] vs. within recommended range: 8.29 [95%CI: 7.30–9.19] vs. excessive: 8.15 [95%CI: 7.55–8.76]; *p* = 0.776), while significant differences were shown among women who carried to term (inadequate: 9.06 [95%CI: 8.57–9.56] vs. within recommended range: 9.08 [95%CI: 8.64–9.48] vs. excessive: 8.08 [95%CI: 7.58–8.59]; *p* = 0.003); during post hoc analyses, significant differences were identified among the inadequate-excessive (*p* = 0.018) and within recommended range-excessive (*p* = 0.007) categories.

The occurrence of previous and current obstetric outcomes and events was also assessed within the context of GWG, stratified by current PTB vs. control status (Table 5); among controls, no relevant associations were noted between previous PTB (*p* = 0.495), or an experience of GHT (*p* = 0.256) or GDM (*p* = 0.589) and GWG. Similarly, among current PTBs, no associations were shown among GHT (*p* = 0.705) or GDM (*p* = 0.721) and GWG; on the other hand, there was weak evidence against the null hypothesis (*p* = 0.090) when comparing previous occurrence of PTB and GWG status, where women outside the recommended weight gain range were substantially more common among those with previous PTB experience. Irrespective of GDM, the highest proportion of mothers both in the case group (73.0%) and the control group (64.0%) belonged outside of the “within recommended range” category (Table 5).

The consumption frequency of various beneficial and harmful foodstuffs—included in the 7-item dietary index was compared within the context of GWG, stratified by case vs. control status (Table 6): among women with PTB, no relevant associations were noted between different GWG groups and the frequency of consuming vegetables (*p* = 0.333), fish (*p* = 0.894), fast foods (*p* = 0.277), salty snacks (*p* = 0.795), sweets (*p* = 0.483) and soft drinks (*p* = 0.513), respectively; while there was a tendency shown about daily fruit consumers being more likely among PTBs outside the recommended GWG (*p* = 0.090). Among controls, no relevant associations were noted between different GWG groups and the frequency of consuming vegetables (*p* = 0.133), fruits (*p* = 0.730), fish (*p* = 0.413), salty snacks (*p* = 0.484), sweets (*p* = 0.191) and soft drinks (*p* = 0.117), respectively; in comparison, those consuming fast foods more frequently were more likely in the “excessive” GWG category (*p* = 0.013) (Table 6).

The distribution of GWG categories among different age groups of pregnant women is shown in Table 7: no notable differences were shown either among women experiencing PTB or controls (*p* = 0.102 and *p* = 0.758, respectively). No association was identified between women’s level of educational attainment and likelihood of being outside the recommended GWG range, either among cases (lower: 86.9% vs. higher: 75.0%; *p* = 0.346) or controls (lower: 70.2% vs. higher: 65.0%; *p* = 0.716).

Both among PTB cases and controls, those women who were more likely outside the recommended GWG range and did not adhere to the low-sodium diet (PTBs: 9.0% vs. 27.0%; *p* = 0.015; controls: 6.0% vs. 19.5%; *p* = 0.031). On the other hand, no relevant associations were shown between GWG categories and adherence to the GDM diet, either in respect to cases (*p* = 0.309) or controls (*p* = 0.224).

The association between various lifestyle factors during gestation and GWG is summarized in Table 8. No associations were shown between GWG groups and smoking (*p* = 0.357) or alcohol consumption (*p* = 0.404) in the PTB group, while there was a higher tendency of being in the recommended GWG range if one participated in physical activity during pregnancy (*p* = 0.088). Among controls, no associations were shown between GWG groups and smoking (*p* = 0.509) or participating in physical activity during pregnancy (*p* = 0.224), while there was a significantly higher chance of being in the recommended GWG range if one consumed alcohol during pregnancy (*p* = 0.045).

Table 9 summarizes the results of multivariable LR analyses (Model 1), where experience of PTB was the main outcome variable, while the participants’ age group, pre-pregnancy BMI category, GWG category, educational attainment, physical activity, smoking and alcohol consumption during pregnancy, GDM, GHT, and dietary index score were included as predictors. Of note, women within the recommended range had a 61.0% lower (aOR: 0.39; 95% CI: 0.18–0.87; *p* = 0.020), while those presenting with GHT had a 243.0% higher (aOR: 3.43; 95% CI: 1.44–8.19; *p* = 0.005) odds of PTB, according to the results of the LR model.

Multivariable LR analyses (Model 2) were also performed to assess the relationship between excessive GWG (as the main outcome variable), and participants’ age group, pre-pregnancy BMI category, educational attainment, physical activity during pregnancy, PTB experience (i.e., case vs. control status) and dietary index score (as predictors), respectively; the results of these analyses are summarized in Table 10. Women who were in the normal (aOR: 4.81; 95% CI: 1.01–22.99; *p* = 0.049), overweight (aOR: 12.38; 95% CI: 2.44–62.81; *p* = 0.002) or obese (aOR: 8.89; 95% CI: 1.71–46.22; *p* = 0.009) pre-pregnancy weight categories were more likely to have excessive GWG; on the other hand, the likelihood of excessive GWG decreased by 17% for every incremental increase in the dietary risk score, according to the results of the LR model (*p* = 0.011) (Table 10).

## 4. Discussion

The high prevalence of PTB is a major public health concern, as complications of prematurity are the leading causes of death in children under five years of age [1]. Due to the resulting healthcare and societal burden, PTB remains a major clinical concern, therefore identifying relevant PTB risk factors and developing cross-cutting, integrated preventive strategies remain a critical priority. In our single-center observational case–control study, the relationship between PTB and GWG was examined at a tertiary obstetric CC in the Southern region of Hungary, and we explored whether maternal dietary habits and lifestyle factors show noteworthy associations with GWG among women delivering preterm versus at term.

In our sample, more than one-third of women—both among the cases and controls—were overweight or had obesity pre-pregnancy, and the distribution of BMI categories before conception also did not differ considerably. These proportions are similar to data of the latest (2019) European Health Interview Survey (EHIS), where 62.8% of Hungarian women—aged 15–34 years—were self-reporting being within the normal weight range, while of 19.4% of respondents were overweight and 10.3% had obesity, respectively [46].

Previous PTB was significantly more common among current PTB cases than in controls (*p* < 0.001), consistent with prior studies identifying prior PTB as one of the strongest predictors of recurrence [47]; in addition, GHT was also more common among cases (*p* = 0.003), in line with existing evidence linking hypertensive disorders to PTB and other adverse neonatal outcomes [12,25]. In contrast, we did not observe any relevant differences between cases and controls within our sample, in regard to smoking, alcohol consumption rates, or the prevalence of GDM.

As reported by earlier studies, inadequate GWG and pre-pregnancy BMI (underweight or overweight/obesity) may increase the risk of PTB [25,26,27]. Our findings partly support these observations: almost half of the women in the case group had inadequate weight gain (49.0% vs. 26.8% in controls), whereas excessive GWG was less frequent among PTB cases (21.9% vs. 38.4%). This pattern suggests that insufficient GWG may be more strongly related to PTB in this sample, while excessive GWG was also highly prevalent among term births.

We assessed the maternal diet using a simple 7-item dietary index [43], covering the consumption frequency of vegetables, fruits, fish, soft drinks, salty snacks, fast foods, and sweets. Women in the control group reported slightly healthier dietary behaviors than PTB cases, with higher proportions of daily vegetable and fruit consumption; however, the mean dietary index scores—used as a cumulative measure—did not show significant differences between groups, and only about one-third of women overall met the threshold to be considered for having a “healthy” diet, according to our pre-defined criteria (index ≥ 10 points) [43]. Interestingly, a consistent tendency of higher dietary index scores was observed among older pregnant women in our sample.

Among controls, lower dietary index scores and more frequent fast-food intake were associated with excessive GWG, whereas similar associations were not observed among PTB cases. These findings are consistent with earlier reports that diets rich in ultra-processed, energy-dense foods, fats, and sugars are linked to excessive GWG and suboptimal infant anthropometric outcomes [27,34,35,36,42], suggesting that diet quality and its constituents may play a particularly important role for preventing excessive GWG in term pregnancies.

In contrast, lifestyle factors, such as smoking and alcohol consumption, were infrequent and showed no clear pattern with GWG in women with PTB, although previously, several studies have reported the existence of an association [16,17,19]. The lack of observing this association with PTB may be due to the high proportion of women reporting healthy behaviors, and the small number of smokers and consumers of alcohol in our sample. Physical activity during pregnancy tended to be associated with recommended GWG among women with PTB, but no consistent effect was seen in controls, and we could not confirm a protective effect on PTB risk, as suggested by previous studies [21].

The multivariable LR analysis (Model 1) confirmed the association between PTB and GWG and GHT. GWG being within the recommended range decreased, while the presence of GHT increased the likelihood of PTB. Furthermore, Model 2 found that women who were in the normal, overweight, or obese pre-pregnancy weight categories were more likely to have excessive GWG, and the risk of excessive GWG decreased with the increase in the dietary risk score, i.e., with healthier dietary habits.

A notable finding was the association between self-reported adherence to a low-sodium diet and GWG. Women who did not follow a low-sodium diet were more often outside the recommended GWG range, compared to those who reported following a low-sodium diet, in both PTB cases (27.0% vs. 9.0%) and controls (19.5% vs. 6.0%). Sodium restriction is widely thought to reduce water retention and thus, lower body weight or attenuate weight gain, primarily through reductions in extracellular fluid volume rather than changes primarily in energy metabolism [48,49]. Although low-sodium diets are often prescribed to prevent hypertensive disorders [50,51]. A self-reported low-sodium diet showed no correlation with GHT in our population, but it did associate strongly with pre-pregnancy BMI, indicating that women who were overweight and women with obesity were more likely to report following a low-sodium diet. A plausible explanation may be that women were advised to restrict salt intake due to a perceived increased risk of GHT or preeclampsia, or because of excessive GWG, which raised suspicion of fluid retention and elevated cardiometabolic risk. However, we did not quantify actual sodium intake, and the self-reported, observational nature of this measure does not allow us to determine precisely whether sodium restriction itself influenced GWG or not.

The results of the present study need to be interpreted with caution, within the context of its limitations and potential sources of bias, including the retrospective data collection methodology, and the use of post-partum self-report on several key exposures of the study, which may potentially introduce recall bias and measurement inaccuracies. Mothers with PTB may recall pregnancy-related behaviors differently compared with mothers of term infants. Additionally, dietary habits were assessed using a simple 7-item dietary index; therefore, it reflects general dietary behavior rather than a comprehensive quantification of nutritional intake of relevant macro- and micronutrients, respectively. Furthermore, our observational case–control study was conducted at a single, tertiary-center regional health facility in Hungary, including a relatively low sample size (and thus, statistical power), which may limit statistical reliability, particularly in stratified analyses with small counts within subgroups, increase the risk of type II error, and may have introduced selection bias, thereby affecting the external validity of our findings. Finally, some questions pertaining to lifestyle and dietary habits were qualitative/categorical (e.g., whether the participant followed the low-sodium diet or not), without actually measuring the salt intake of the participants. This method of data collection from the participants may reflect medical advice received or perceived health risks, rather than actual dietary sodium intake. Additionally, the lack of detailed information on portion sizes and energy intake may have contributed to misclassification bias. Therefore, the observed associations should be interpreted with caution, as they may reflect reverse causation or confounding by clinical indication.

Despite the limitations of our study, our findings have notable strengths as well, including its matched case–control study design, to reduce confounding by main socio-demographic and pregnancy-related variables, the use of internationally accepted IOM guidelines to assess GWG categories, and the evaluation of the quality of maternal diet using a multidimensional dietary index scale. The high prevalence of GWG below or above guideline-recommended ranges in both PTB and term pregnancies suggests that closer monitoring and counseling on managing gestational weight should be integrated into routine antenatal care. Our results support the role of inadequate GWG as a potential marker of PTB risk, and emphasize the need to optimize both pre-pregnancy BMI and GWG as modifiable determinants of maternal and neonatal health outcomes. The patterns observed among term controls, linking frequent fast-food consumption and lower dietary scores to excessive GWG, highlight the importance of dietary interventions, focusing not only on GWG, but also on diet quality and the reduction in ultra-processed, energy-dense foods. Future studies with larger, prospective cohorts and more detailed, objective assessment of diet, physical activity, and sodium intake are needed to clarify the causal pathways between maternal nutrition, GWG, and PTB.

## 5. Conclusions

In this observational case–control study, we found that inadequate GWG was more common among women who delivered preterm, whereas excessive GWG was highly prevalent among term births. Pre-pregnancy overweight and obesity were frequent in both groups. Only about one-third of women reported a “healthy” dietary pattern according to our dietary index. Among term pregnancies, more frequent fast-food consumption was associated with excessive GWG, suggesting that diet quality is an important target for preventing excessive GWG. Self-reported low-sodium diet was related to higher pre-pregnancy BMI, but its association with GWG remains uncertain, due to the lack of quantitative sodium intake measurements.

In summary, our findings suggest that GWG and diet quality should be considered key targets in antenatal care, and that routine weight and nutrition counseling may help reduce PTB and related complications.

## Figures and Tables

**Table 1:** The 7-item dietary index scale utilized in our study [43].

Dietary Habits	Index Score Attained
Vegetable consumption	
Daily	2
Weekly	1
Less frequently	0
Fruit consumption	
Daily	2
Weekly	1
Less frequently	0
Fish consumption	
Weekly	1
Less frequently	0
Fast foods	
Monthly or never	1
More frequently	0
Salty snacks	
Monthly or never	2
Weekly	1
Daily	0
Sweets	
Monthly or never	2
Weekly	1
Daily	0
Soft drinks	
Monthly or never	2
Weekly	1
Daily	0
Maximum total dietary index score	12

**Table 2:** Recommendations for total weight gain at 40 weeks by pre-pregnancy BMI ^1^ according to the U.S. 2009 Institute of Medicine [29].

Pre-Pregnancy BMI ^1^	BMI ^1^ (kg/m^2^)	Total Weight Gain (kg)
Underweight	<18.5	12.5–18.0
Normal weight	18.5–24.9	11.5–16.0
Overweight	25.0–29.9	7.0–11.5
Obese (includes all classes)	≥30.0	5.0–9.0

^1^ BMI: body mass index.

**Table 3:** Demographic characteristics and anamnestic data of women with PTB ^1^ and controls.

Variables of Interest		Total	Cases	Controls	*p*-Value *
		*n* (%)	*n* (%)	*n (%)*	
Age-group (years)	18–24	34 (11.3)	12 (12.0)	22 (11.0)	0.965
	25–34	158 (52.7)	52 (52.0)	106 (53.0)	
	≥35	108 (36.0)	36 (36.0)	72 (36.0)	
Level of educational attainment	lower	122 (41.5)	47 (49.5)	75 (37.7)	0.055
	higher	172 (58.5)	48 (50.5)	124 (62.3)	
Relationship status	single	7 (2.4)	3 (3.0)	4 (2.0)	0.603
	in a relationship	290 (97.6)	97 (97.0)	193 (98.0)	
Residence	county town	134 (45.0)	38 (38.4)	96 (48.2)	0.224
	town	89 (29.9)	35 (35.4)	54 (27.1)	
	village	75 (25.2)	26 (26.3)	49 (24.6)	
Pre-pregnancy BMI ^2^ groups	underweight	15 (5.1)	5 (5.1)	10 (5.0)	0.882
	normal weight	171 (57.6)	55 (56.1)	116 (58.3)	
	overweight	59 (19.5)	22 (22.4)	37 (18.6)	
	obese	52 (17.5)	16 (16.3)	36 (18.1)	
Smoking during pregnancy	no	282 (94.0)	91 (91.0)	191 (95.5)	0.122
	yes	18 (6.0)	9 (9.0)	9 (4.5)	
Physical activity during pregnancy	no	155 (52.4)	100 (50.5)	55 (56.1)	0.362
	yes	141 (47.6)	98 (49.5)	43 (43.9)	
Alcohol consumption during pregnancy	no	271 (91.2)	94 (94.9)	177 (89.4)	0.110
	yes	26 (8.8)	5 (5.1)	21 (10.5)	
History of PTB ^1^	no	142 (83.0)	41 (68.3)	101 (91.0)	**<0.001**
	yes	29 (17.0)	19 (31.7)	10 (9.0)	
GHT ^3^	no	264 (89.2)	80 (81.6)	184 (92.9)	**0.003**
	yes	32 (10.8)	18 (18.4)	14 (7.1)	
GDM ^4^	no	251 (84.8)	80 (81.6)	171 (86.4)	0.286
	yes	45 (15.2)	18 (18.4)	27 (13.6)	
GDM ^4^ diet during pregnancy	no	65 (51.6)	21 (44.7)	44 (55.7)	0.231
	yes	61 (48.4)	26 (55.3)	35 (44.3)	
Low-sodium diet	no	96 (76.8)	36 (76.6)	60 (76.9)	0.967
	yes	29 (23.2)	11 (23.4)	18 (23.1)	

^1^ PTB: preterm birth; ^2^ BMI: body mass index; ^3^ GHT: gestational hypertension; ^4^ GDM: gestational diabetes mellitus; * based on χ^2^-test or Fisher’s exact tests; *p*-values < 0.05 were denoted in **boldface.**

**Table 4:** The characteristics of maternal dietary habits during pregnancy among women with PTB ^1^ and controls.

Dietary Habits *	Total	Cases	Controls	*p*-Value **
	*n* (%)	*n* (%)	*n* (%)	
Vegetable consumption				
Daily	233 (78.5)	68 (69.4)	165 (82.9)	**0.025**
Weekly	61 (20.5)	29 (29.6)	32 (16.1)	
Less frequently	3 (1.0)	1 (1.0)	2 (1.0)	
Fruit consumption				
Daily	237 (81.4)	73 (76.0)	164 (84.1)	**0.043**
Weekly	50 (17.2)	23 (24.0)	27 (13.8)	
Less frequently	4 (1.4)	0 (0.0)	4 (2.1)	
Fish consumption				
Weekly	106 (35.6)	37 (37.0)	69 (34.8)	0.714
Less frequently	192 (64.4)	63 (63.0)	129 (65.2)	
Fast foods				
Monthly or never	228 (77.0)	72 (72.0)	156 (79.6)	0.142
More frequently	68 (23.0)	28 (28.0)	40 (20.4)	
Salty snacks				
Monthly or never	151 (51.0)	48 (48.5)	103 (52.3)	0.653
Weekly	126 (42.6)	43 (43.4)	83 (42.1)	
Daily	19 (6.4)	8 (8.1)	11 (5.6)	
Sweets				
Monthly or never	44 (15.0)	16 (16.3)	28 (14.3)	0.506
Weekly	164 (55.8)	50 (51.0)	114 (58.2)	
Daily	86 (29.3)	32 (32.7)	54 (27.6)	
Soft drinks				
Monthly or never	174 (58.6)	58 (58.0)	116 (58.9)	0.291
Weekly	95 (32.0)	29 (29.0)	66 (33.5)	
Daily	28 (9.4)	13 (13.0)	15 (7.6)	

^1^ PTB: preterm birth; * possible options were based on the dietary index scale [43]; ** based on χ^2^-test or Fisher’s exact tests; *p*-values < 0.05 were denoted in **boldface.**

**Table 5:** Association between previous and present obstetric events and gestational weight gain among women with PTB ^1^ and controls.

Weight Gain Categories	Cases *n* (%)		Controls *n* (%)	
Previous PTB ^1^				
	yes	no	yes	no
Inadequate	4 (25.0)	16 (40.0)	1 (11.1)	24 (24.0)
Within the recommended range	1 (6.3)	9 (22.5)	3 (33.3)	39 (39.0)
Excessive	11 (68.8)	15 (37.5)	5 (55.6)	37 (37.0)
*p*-values *	0.090		0.495	
GHT ^2^				
	yes	no	yes	no
Inadequate	5 (27.8)	28 (38.4)	1 (7.7)	45 (25.1)
Within the recommended range	4 (22.2)	14 (19.2)	4 (30.8)	60 (33.5)
Excessive	9 (50.0)	31 (42.5)	8 (61.5)	74 (41.3)
*p*-values *	0.705		0.256	
GDM ^3^				
	yes	no	yes	no
Inadequate	8 (44.4)	25 (34.2)	8 (30.8)	38 (22.9)
Within the recommended range	3 (16.7)	15 (20.5)	9 (34.6)	55 (33.1)
Excessive	7 (38.9)	33 (45.2)	9 (34.6)	73 (44.0)
*p* -values *	0.721		0.589	

^1^ PTB: preterm birth; ^2^ GHT: gestational hypertension; ^3^ GDM: gestational diabetes mellitus; * based on χ^2^-test or Fisher’s exact tests.

**Table 6:** Consumption frequency of various beneficial and harmful foodstuffs according to gestational weight gain categories in women with PTB ^1^ and controls.

GWG ^2^ Categories		Cases			Controls	
		*n* (%)			*n* (%)	
	Vegetable consumption					
	Daily	Weekly	Less frequently	Daily	Weekly	Less frequently
Inadequate	27 (42.2)	6 (23.1)	1 (100.0)	39 (24.4)	7 (22.6)	0 (0.0)
Within the recommended range	11 (17.2)	6 (23.1)	0 (0.0)	58 (36.3)	6 (19.4)	0 (0.0)
Excessive	26 (40.6)	14 (53.8)	0 (0.0)	63 (39.4)	18 (58.1)	2 (100.0)
*p*-values *	0.333			0.133		
	Fruit consumption					
	Daily	Weekly	Less frequently	Daily	Weekly	Less frequently
Inadequate	28 (40.6)	4 (20.0)	0 (0.0)	39 (24.4)	5 (19.2)	1 (33.3)
Within the recommended range	15 (21.7)	3 (15.0)	0 (0.0)	53 (33.1)	8 (30.8)	0 (0.0)
Excessive	26 (37.7)	13 (65.0)	0 (0.0)	68 (42.5)	13 (50.0)	2 (66.7)
*p*-values *	0.090			0.730		
	Fish consumption					
	Weekly		Less frequently	Weekly		Less frequently
Inadequate	11 (34.4)		23 (37.7)	14 (21.2)		32 (25.2)
Within the recommended range	7 (21.9)		11 (18.0)	26 (39.4)		38 (29.9)
Excessive	14 (43.8)		27 (44.3)	26 (39.4)		57 (44.9)
*p*-values *	0.894			0.413		
	Fast foods					
	More frequently		Monthly or never	More frequently		Monthly or never
Inadequate	8 (34.8)		26 (37.1)	5 (12.5)		41 (27.2)
Within the recommended range	7 (30.4)		11 (15.7)	10 (25.0)		54 (35.8)
Excessive	8 (34.8)		33 (47.1)	25 (62.5)		56 (37.1)
*p*-values *	0.277			**0.013**		
	Salty snacks					
	Daily	Weekly	Less frequently	Daily	Weekly	Less frequently
Inadequate	3 (37.5)	17 (41.5)	13 (30.2)	2 (18.2)	18 (22.5)	26 (25.7)
Within the recommended range	2 (25.0)	8 (19.5)	8 (18.6)	3 (27.3)	23 (28.7)	38 (37.6)
Excessive	3 (37.5)	16 (39.0)	22 (51.2)	6 (54.4)	39 (48.8)	37 (36.6)
*p*-values *	0.795			0.484		
	Sweets					
	Daily	Weekly	Monthly or never	Daily	Weekly	Monthly or never
Inadequate	13 (29.5)	13 (29.5)	8 (53.3)	8 (15.1)	30 (27.0)	8 (29.6)
Within the recommended range	5 (15.6)	9 (20.5)	3 (20.0)	17 (32.1)	40 (36.0)	6 (22.2)
Excessive	14 (43.8)	22 (50.0)	4 (26.7)	28 (52.8)	41 (36.9)	13 (48.1)
*p*-values *	0.483			0.191		
	Soft drinks					
	Daily	Weekly	Monthly or never	Daily	Weekly	Monthly or never
Inadequate	3 (27.3)	12 (44.4)	19 (34.5)	1 (7.1)	16 (24.2)	29 (25.9)
Within the recommended range	1 (9.1)	4 (14.8)	13 (23.6)	3 (21.4)	19 (28.8)	42 (37.5)
Excessive	7 (63.6)	11 (40.7)	23 (41.8)	10 (71.4)	31 (47.0)	41 (36.6)
*p*-values *	0.513			0.117		

^1^ PTB: preterm birth; ^2^ GWG: gestational weight gain; * based on χ^2^-test or Fisher’s exact tests; *p*-values < 0.05 were denoted in **boldface.**

**Table 7:** Distribution of age groups according to gestational weight gain among women with PTB ^1^.

GWG ^2^ Categories	Cases *n* (%)			Controls *n* (%)		
	Age Groups					
	18–24 Years	25–34 Years	≥35 Years	18–24 Years	25–34 Years	≥35 Years
Inadequate	5 (41.7)	11 (23.9)	18 (51.4)	4 (20.0)	24 (23.1)	19 (29.7)
Within the recommended range	1 (8.3)	12 (26.1)	5 (14.3)	5 (25.0)	35 (33.7)	24 (34.3)
Excessive	6 (50.0)	23 (50.0)	12 (34.3)	11 (55.0)	45 (43.3)	27 (38.6)
*p*-value *	0.102			0.758		

^1^ PTB: preterm birth; ^2^ GWG: gestational weight gain; * based on χ^2^-test or Fisher’s exact tests.

**Table 8:** Association of lifestyle factors during gestation and gestational weight gain categories in women with PTB ^1^ and controls.

GWG ^2^ Categories	Cases *n* (%)		Controls *n* (%)	
Smoking during pregnancy				
	yes	no	yes	no
Inadequate	2 (22.2)	32 (38.1)	2 (25.0)	45 (24.2)
Within the recommended range	1 (11.1)	17 (20.2)	4 (50.0)	60 (32.3)
Excessive	6 (66.7)	35 (41.7)	2 (25.0)	81 (43.5)
*p* -value *	0.357		0.509	
Physical activity during pregnancy				
	yes	no	yes	no
Inadequate	10 (25.0)	23 (45.1)	27 (28.1)	19 (19.8)
Within the recommended range	11 (27.5)	7 (13.7)	33 (34.4)	30 (31.2)
Excessive	19 (47.5)	21 (41.2)	36 (37.5)	47 (49.0)
*p* -value *	0.088		0.224	
Alcohol consumption during pregnancy				
	yes	no	yes	no
Inadequate	2 (40.0)	32 (36.8)	4 (19.0)	42 (24.6)
Within the recommended range	2 (40.0)	16 (18.4)	12 (57.1)	52 (30.4)
Excessive	1 (20.0)	39 (44.8)	5 (23.8)	77 (45.0)
*p* -value *	0.404		**0.045**	

^1^ PTB: preterm birth; ^2^ GWG: gestational weight gain; * based on χ^2^-test or Fisher’s exact tests; *p*-values < 0.05 were denoted in **boldface**.

**Table 9:** Model 1: Multivariable logistic regression analyses (outcome variable: PTB ^1^ experience).

Variables	β	SE ^2^	aOR (95% CI) ^3^	*p*-Value
Dietary index (score)	−0.12	0.08	0.89 (0.76–1.03)	0.120
Age groups				
18–24 (Ref.)			1.00	
25–34	0.28	0.55	1.32 (0.48–3.68)	0.596
≥35	0.18	0.08	1.20 (0.41–3.52)	0.738
Pre-pregnancy BMI ^4^ category				
Underweight (Ref.)			1.00	
Normal weight	−0.01	0.62	0.99 (0.29–3.34)	0.991
Overweight	0.13	0.68	1.14 (0.31–4.30)	0.845
Obese	−0.34	0.71	0.71 (0.18–2.65)	0.633
GWG ^5^ categories				
Inadequate (Ref.)			1.00	
Within the recommended range	**−0.92**	**0.39**	**0.39 (0.18–0.87)**	**0.020**
Excessive	−0.59	0.35	0.55 (0.28–1.10)	0.092
Level of educational attainment				
Lower (Ref.)			1.00	
Higher	−0.23	0.31	0.79 (0.42–1.48)	0.466
Physical activity during pregnancy				
No (Ref.)			1.00	
Yes	0.134	0.296	1.14 (0.64–2.04)	0.650
Smoking during pregnancy				
No (Ref.)			1.00	
Yes	0.72	0.61	2.06 (0.62–6.81)	0.236
GDM ^5^				
No (Ref.)			1.00	
Yes	0.62	0.40	1.86 (0.85–4.09)	0.121
GHT ^6^				
No (Ref.)			1.00	
Yes	**1.24**	**0.44**	**3.43 (1.44–8.19)**	**0.005**

^1^ PTB: preterm birth; ^2^ SE: standard error; ^3^ aOR: adjusted odds ratio, CI: confidence interval; ^4^ BMI: body mass index; ^5^ GDM: gestational diabetes mellitus; ^6^ GHT: gestational hypertension; β: regression coefficient; Ref.: reference level during regression analyses; *p*-values < 0.05 were denoted in **boldface.**

**Table 10:** Model 2: Multivariable logistic regression analyses (outcome variable: excessive GWG ^1^).

Variables	β	SE ^2^	aOR (95% CI) ^3^	*p*-Value
Dietary index (score)	**−0.19**	**0.07**	**0.83 (0.72–0.96)**	**0.011**
Age groups				
18–24 (Ref.)			1.00	
25–34	−0.12	0.48	0.88 (0.35–2.21)	0.792
≥35	−0.43	0.49	0.65 (0.25–1.73)	0.390
Pre-pregnancy BMI ^4^ category				
Underweight (Ref.)			1.00	
Normal weight	**1.57**	**0.79**	**4.81 (1.01–22.99)**	**0.049**
Overweight	**2.51**	**0.82**	**12.38 (2.44–62.81)**	**0.002**
Obese	**2.18**	**0.84**	**8.89 (1.71–46.22)**	**0.009**
Level of educational attainment				
Lower (Ref.)			1.00	
Higher	−0.155	0.29	0.86 (0.49–1.51)	0.580
Physical activity during pregnancy				
No (Ref.)			1.00	
Yes	−0.05	0.271	0.95 (0.56–1.61)	0.846
PTB ^5^ experience				
No (Ref.)			1.00	
Yes	−0.14	0.28	0.87 (0.49–1.53)	0.618

^1^ GWG: gestational weight gain; ^2^ SE: standard error; ^3^ aOR: adjusted odds ratio, CI: confidence interval; ^4^ BMI: body mass index; ^5^ PTB: preterm birth; β: regression coefficient; Ref.: reference level during regression analyses; *p*-values < 0.05 were denoted in **boldface.**

## Data Availability

The dataset is available from the Corresponding author on reasonable request.

## Supplementary Materials

The following supporting information can be downloaded at: https://www.mdpi.com/article/10.3390/nu18091403/s1, Supplementary Material S1: STROBE checklist.

## Abbreviations

The following abbreviations are used in this manuscript:

aOR	adjusted odds ratio
BMI	Body Mass Index
CC	Clinical Center
CDC	Centers for Disease Control and Prevention
CI	confidence interval
DALYs	disability-adjusted life-years
EHIS	European Health Interview Survey
GBD	Global Burden of Disease, Injuries and Risk Factors
GDM	gestational diabetes mellitus
GHT	gestational hypertension
GWG	gestational weight gain
IOM	Institute of Medicine
LBW	low birth weight
LGA	large-for-gestational age
LR	logistic regression
PTB	preterm birth
SDI	Socio-Demographic Index
SE	standard error
SGA	small-for-gestational age
SPSS	Statistical Package for Social Sciences
US	United States
WHO	World Health Organization
β	coefficient
