# Supplementary material for: Maternal Diet, Lifestyle Factors, and Gestational Weight Gain: A Single-Center Case–Control Study in Hungary
Source: Nutrients. 2026 Apr 29;18(9):1403. doi: 10.3390/nu18091403 (PMC13164820; doi:10.3390/nu18091403)
Supplement: Supplementary file 1 [file nutrients-18-01403-s001.zip › nutrients-4198346-supplementary.pdf]

Item No.	Recommendation	Page No.
1	Indicate the study’s design with a commonly used term in the title or abstract	1
2	Explain the scientific background and rationale for the investigation	2-3
3	State specific objectives, including any prespecified hypotheses	3-4
4	Present key elements of study design early in the paper	4
5	Describe the setting, locations, and relevant dates	4
6	Give the eligibility criteria and the sources and methods of selection of participants	5
7	Clearly define all outcomes, exposures, predictors, potential confounders, and effect modifiers	5-7
8	For each variable of interest, give sources of data and details of methods of assessment	5-7
9	Describe any efforts to address potential sources of bias	6
10	Explain how the study size was arrived at	4-5
11	Explain how quantitative variables were handled in the analyses	7
12	Describe all statistical methods, including those used to control for confounding	7
13	Give numbers of individuals at each stage of study	4, 8
14	Give characteristics of study participants	7-8
15	Report numbers of outcome events or summary measures	8-13
16	Give unadjusted estimates and, if applicable, confounder-adjusted estimates	14-15
17	Report other analyses done (e.g., subgroups, interactions)	not relevant
18	Summarise key results with reference to study objectives	16-17
19	Discuss limitations of the study	17
20	Give a cautious overall interpretation of results	16-18
21	Discuss the generalisability (external validity) of the study results	18
22	Give the source of funding and the role of the funders	18
